# Predicting odor from molecular structure: a multi-label classification approach

**DOI:** 10.1038/s41598-022-18086-y

**Published:** 2022-08-16

**Authors:** Kushagra Saini, Venkatnarayan Ramanathan

**Affiliations:** 1 Department of Chemical Engineering, Indian Institute of Technology (Banaras Hindu University, Varanasi, U.P. 221005 India; 2 Department of Chemistry, Indian Institute of Technology (Banaras Hindu University), Varanasi, U.P. 221005 India

**Keywords:** Chemical biology, Computational biology and bioinformatics, Mathematics and computing

## Abstract

Decoding the factors behind odor perception has long been a challenge in the field of human neuroscience, olfactory research, perfumery, psychology, biology and chemistry. The new wave of data-driven and machine learning approaches to predicting molecular properties are a growing area of research interest and provide for significant improvement over conventional statistical methods. We look at these approaches in the context of predicting molecular odor, specifically focusing on multi-label classification strategies employed for the same. Namely binary relevance, classifier chains, and random forests adapted to deal with such a task. This challenge, termed quantitative structure–odor relationship, remains an unsolved task in the field of sensory perception in machine learning, and we hope to emulate the results achieved in the field of vision and auditory perception in olfaction over time.

## Introduction

For decades, scientists from various disciplines have been searching for an olfactory classification system on a psychological or physiochemical basis^1^. A robust model that can accurately predict odor would cut down significantly on the time and capital spent in formulation, extraction, and production of new odors for which there is substantial commercial demand. There is also an incentive to find substitutes for odorants that are environmentally hazardous or very scarce. For example, sandalwood scent is derived from santalol, and in an effort to synthetically reproduce the scent, chemist Jacques Vailliant spent more than a year creating structural variations of santalol by changing the ring and branch structures with very little success^2^.

The biological process of olfaction is characterized by the odorant molecules entering the nasal cavity where odor perception is due to the interaction of volatile compounds with the olfactory receptor neurons (ORNs) that lie in the olfactory epithelium, which occupies a 3.7 cm zone in the upper part of the nasal cavity^3^. Humans have around 12 million ORNs in each epithelium (right and left); as the olfaction system is bilateral, there are two of each structure^3^.

Broadly there are two general approaches to odor classification: -

1. By featurizing of the molecular properties/Structure of odorous compound-This approach tries to establish a link between the molecular/structural properties like molecular vibration, molecular weight, molecule shape, electron donor, acid–base, and other physicochemical parameters and perceived odor on the principle of capturing some global relation between a set of features and target variable^4^. For example, it is common knowledge that molecules with an ester functional group usually have a fruity and floral smell. The vibrational spectrum can also serve as a proxy for the structure of a molecule and form the feature space^5^.
2. As features of the sensory percept- This school of thought argues that odor is merely a psychological construct and therefore entirely subjective. The basis for classification then becomes the verbal odor descriptions used by subjects to describe a particular odor. A review of a large body of research reported that many of the proposed perception-based classification systems are vague or even contradictory^6^.This can be attributed to differences in subjects’ vocabulary, sensitivity to odor with age, cultural experience, etc. It is now, therefore, rarely used in scientific literature and is more or less obsolete.

Another less frequently used approach focuses on mouse olfactory sensory neurons (OSNs) where receptor activity through a calcium signal forms the basis for odor instead of subjective descriptors from human subjects^7^.

### QSOR modelling in the past

As a subdomain of the molecular property prediction problem (also called QSAR or quantitative structure–activity relationships)^8^, interest has been revived in QSOR over the last decade, with machine learning algorithms becoming more and more complex, and especially with breakthroughs in the field of deep learning and neural networks^9^.

Early implementations of neural networks for QSOR were very shallow and modelled on overly small odor and sample spaces (rarely more than 100 molecules were used and categories of odor to classify numbered between one and 10)^10–12^.While the exact structure of odor space and its dimensions is still an area of active research, it is well established that the magnitude of a global odor space, if it exists, is at least in the100s.

### Challenges in QSOR

**Figure 1:**
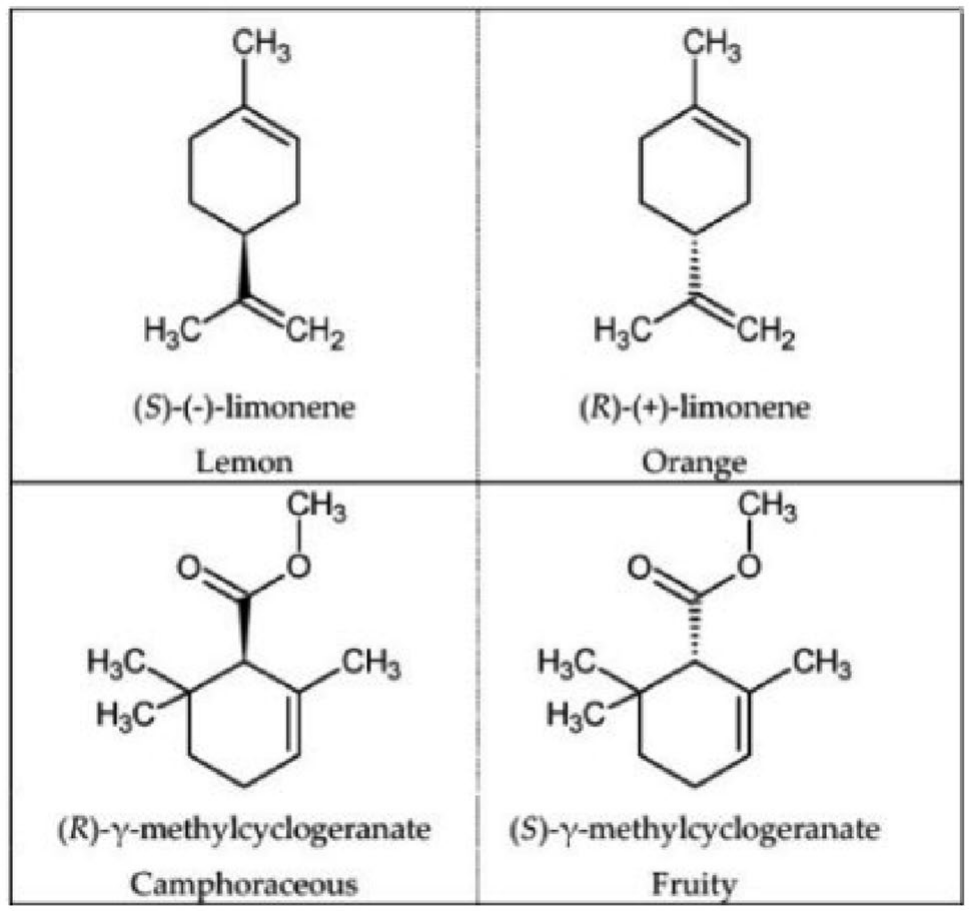
Examples of enantiomeric compounds with dissimilar odors.

**Figure 2:**
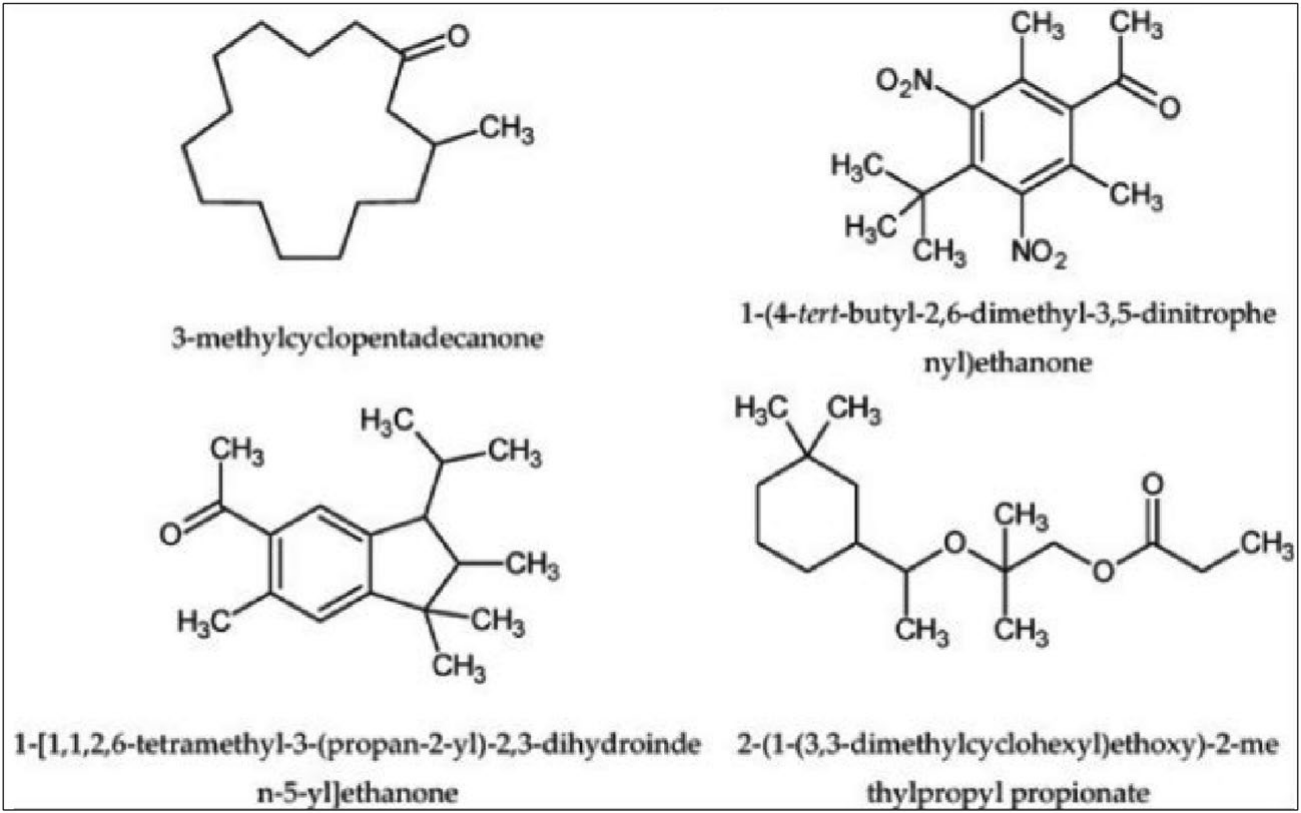
Examples of odorants with different structures but similar odor (musk).

**Figure 3:**
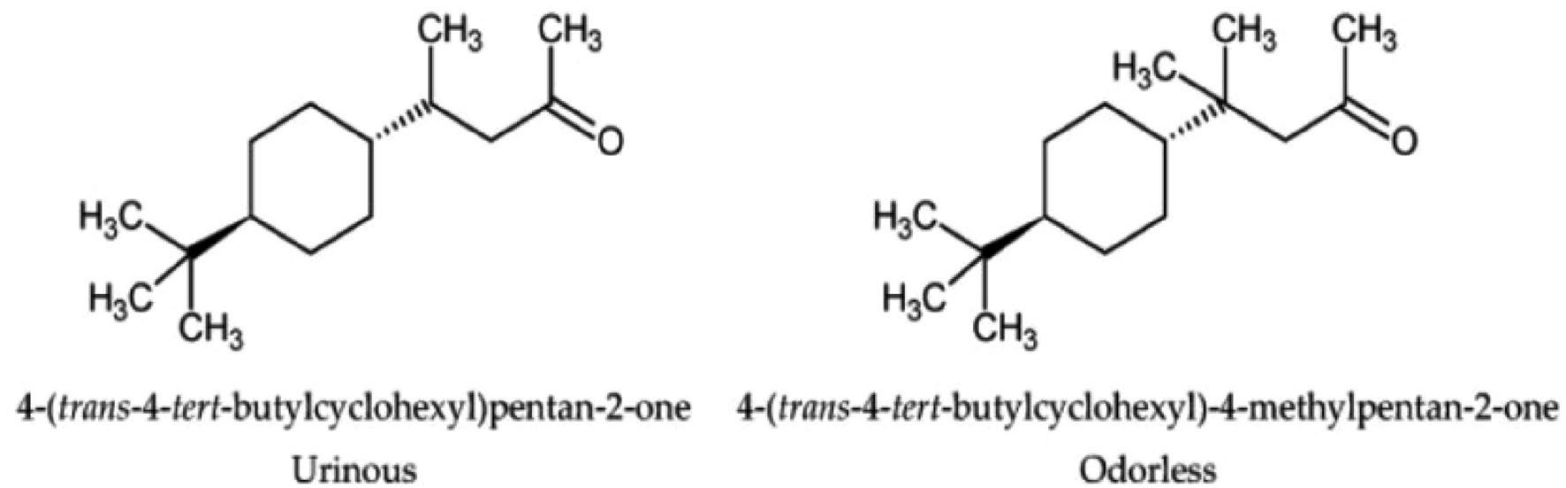
Example of the changing odorant character of a compound with slight structural modification.

The main hurdle in QSOR is the drastic change in odor caused by very small changes in the structure or functional group of a molecule. Early attempts at generating a set of rigid empirical odor rules on the basis of molecular substituent, intramolecular distances, and other molecular properties for odor prediction were immediately broken any time a new odorant molecule was discovered and thus were full of exceptions. Here are some of the examples which defied the odor rules:

1. Enantiomeric compounds, also known as optical isomers, have the same chemical functions and are structurally close, but only as few as 5% of enantiomer couples have a similar smell. Two such examples are shown in Fig. 1^13^.
2. Structurally different organic compounds having a similar smell, for example, musk-related odors shown in Fig. 2^14^.
3. Besides changing the odor, a small structural change to a molecule may also cause a decrease in odor intensity as shown in Fig. 3.

## Methods

### Forming an integrated dataset

Two separate expertly labeled odor datasets were used during the course of this study, namely Leffingwell PMP 2001 and the training data made available during the “learning to smell challenge” by Firmenich. The Leffingwell dataset was originally curated for researching olfaction and all the odorant molecules in it were labeled with one or more odor descriptors, hand-picked by olfactory experts (usually a practicing perfumer). In order to come up with an integrated dataset that could be directly fed to our model, merging of the aforementioned datasets using a common schema was required along with the filtering of duplicate odorous molecules.

**Figure 4:**
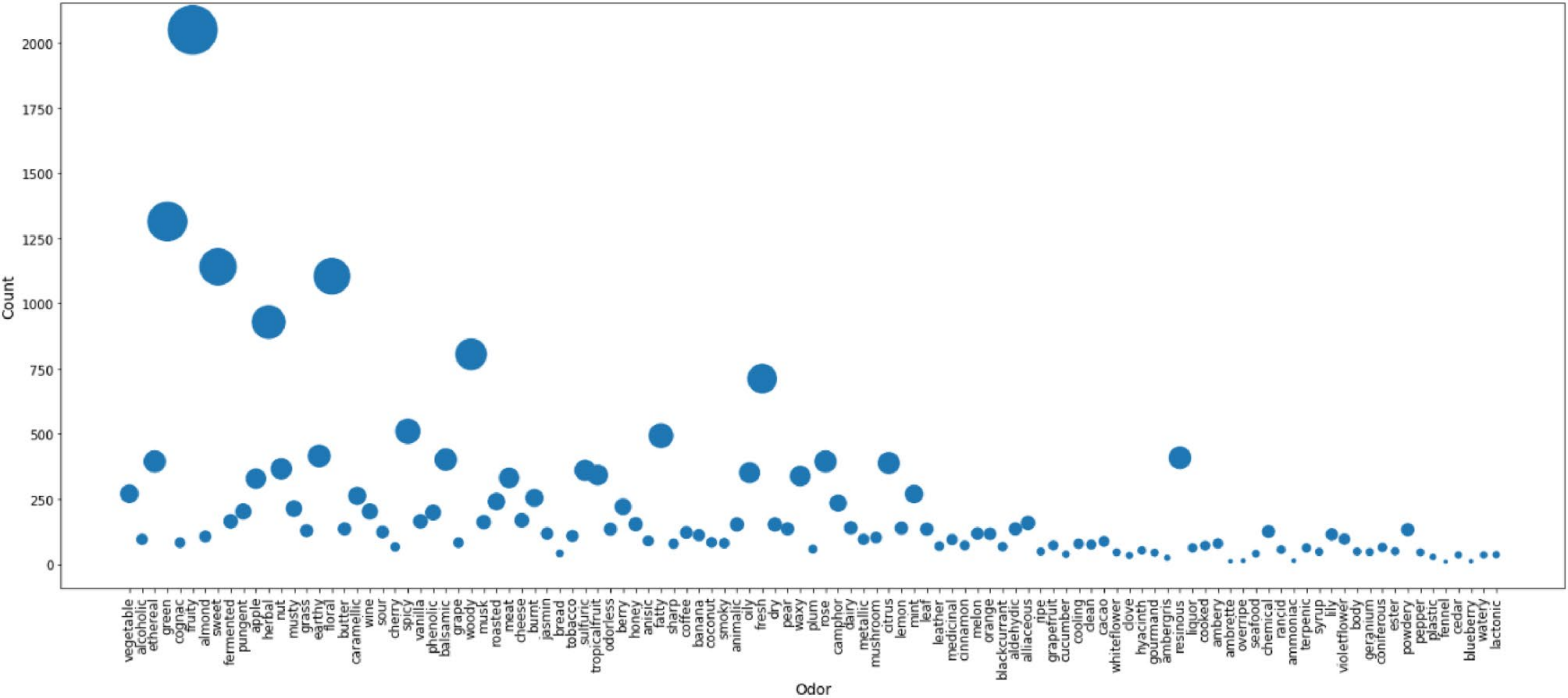
A scatter plot representing the number of samples in each of the 109 unique odor classes.The size of each point in the plot is proportional to the magnitude of the number of samples in that odor class.

Firmenich's dataset had a total of 4704 molecules and a vocabulary of 109 unique odor descriptors, while the PMP dataset contained 3523 molecules and 113 unique odor descriptors. We observed that 61 odor descriptors were common in both the datasets and upon closer inspection, it was found that some odor descriptors were identical semantically but different syntactically ('black currant' vs. 'blackcurrant,' 'leafy' vs. 'leaf' etc.). A similarity-based string search using the fuzzywuzzy module was done to make a list of such odor descriptors; we manually removed any pairs which were not deemed to be referring to the same odor. Further transformation of the pair of odor descriptors to fit in with Firmenich's vocabulary was done which resulted in a total of 75 intersecting odor descriptors. The final dataset formed by merging both the datasets had a total of 7374 molecule samples and 109 unique odor classes with varying sample counts (Fig. 4).The highest number of samples were associated with the fruity class at a count of 2050 samples, while the fennel class had the lowest associated sample count with 9 samples. A standard training/test split of 80:20 percent was set for our model evaluation and a five-fold cross-validation was conducted on the training set.

### Exploring label imbalance

Multi-label datasets (MLDs) typically have heavy label imbalance. To verify this a couple of label imbalance metrics by the name of MeanIR and IRLbl were computed along with plotting a histogram of odor labels to visually infer if any such imbalances remained. Further, class_weight hyper parameter for the models was set to “balanced” to tackle class imbalances within our dataset.

### Exploring label correlation

Where a multi-class task would have only one odor label associated with the molecule out of 109 unique odors in the dataset, a multi-label task has one or more than one odor labels associated with a molecule out of 109 unique odors.

For example.

**Table:** 

Multi-class		Multi-label	
Molecule	Odor	Molecule	Odor
CSC#N	['alliaceous']	CCC(=O)O	['pungent', 'sour', 'dairy']

Generally, complete independence between the labels is assumed. However, most researchers highlight the importance to take into account label dependency information^15^. For example, a ‘fruity’ label may be more likely to occur with the label ‘apple’ based on linguistic similarity. A co-occurrence matrix (109 × 109, for 109 unique odors in the integrated dataset) where i’th row and j’th column represents the frequency of the co-occurrence of the two labels was built to gauge label dependency up to second degree. To get a more global knowledge of any such label correlation, we ran the Louvain community detection algorithm on the odor labels which attempts to optimize modularity; a measure for the quality of partition between communities of nodes.

### Featurizing molecules

To get a meaningful numerical representation of odor molecules which to be fed to our model, we used three traditional featurization techniques:- Mordred, Morgan, and daylight fingerprinting. The first of the three was generated using Mordred descriptor calculator^16^ while Rdkit was used for the other two.

### Pre-processing

Some columns from the feature space were dropped due to a large percentage of missing values. The remaining missing values were imputed using a KNN Imputer. Furthermore, each label set was converted into a 109-length bit vector; 1 denoting the presence of the label and 0 denoting its absence with the associated molecule.

### Training machine learning models

We used a random forest classifier with multi-label support^17^ in the scikit-learn library as our baseline model. Further, we use two different model approaches for chaining random forest models together:- Binary Relevance and Classifier chains^18^.

### Using evaluation metrics to validate model performance

To measure multi-label classifiers, we averaged the classes. We used the micro-averaging method where the individual true positives, false positives, and false negatives of the system for different label sets were averaged. The micro-averaged F1-Score represented the harmonic mean of micro averaged recall and micro averaged precision.

## Results and discussion

**Figure 5:**
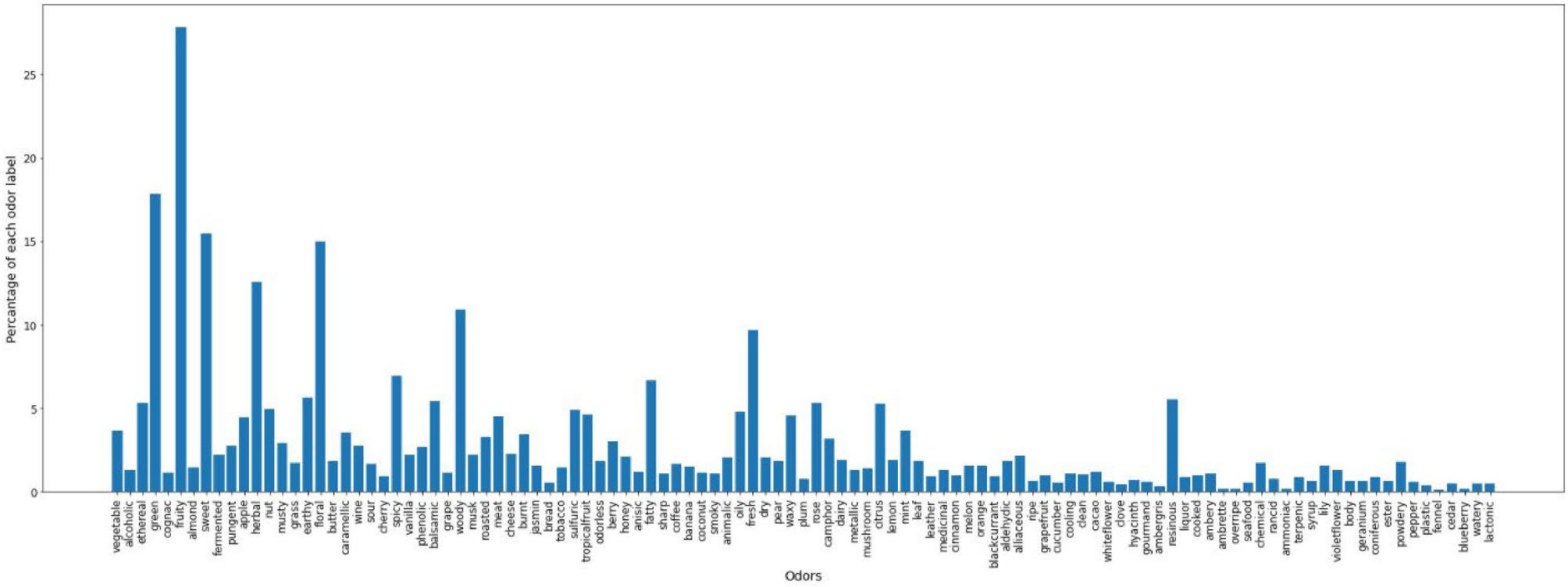
Label imbalance with the fruity label occurring approximately 27% of the time as opposed to fennel occurring less than 1% times in our data.

A preliminary histogram plot (Fig. 5) revealed that fennel has the least occurrence in our dataset while fruity is the highest occurring label. As apparent, the distribution is highly skewed implying a heavy label imbalance. This imbalance was further verified by computing the MeanIR (29.169). The disparity between the absolute frequency of the top 10 most frequently occurring and least frequently occurring odor labels make the skewness more apparent in Table 1.

**Table 1:** Top 10 most frequently (from ‘Fruity' till ‘Earthy') and least frequently (from ‘Fennel' till ‘Watery') occurring Odors respectively.

Odor	No of associated samples
Fruity	2050
Green	1314
Sweet	1140
Floral	1104
Herbal	928
Woody	805
Fresh	712
Spicy	510
Fatty	493
Earthy	415
Fennel	9
Ambrette	11
Blueberry	11
Overripe	13
Ammoniac	13
Ambergris	24
Plastic	27
Clove	33
Cedar	35
Watery	35

**Figure 6:**
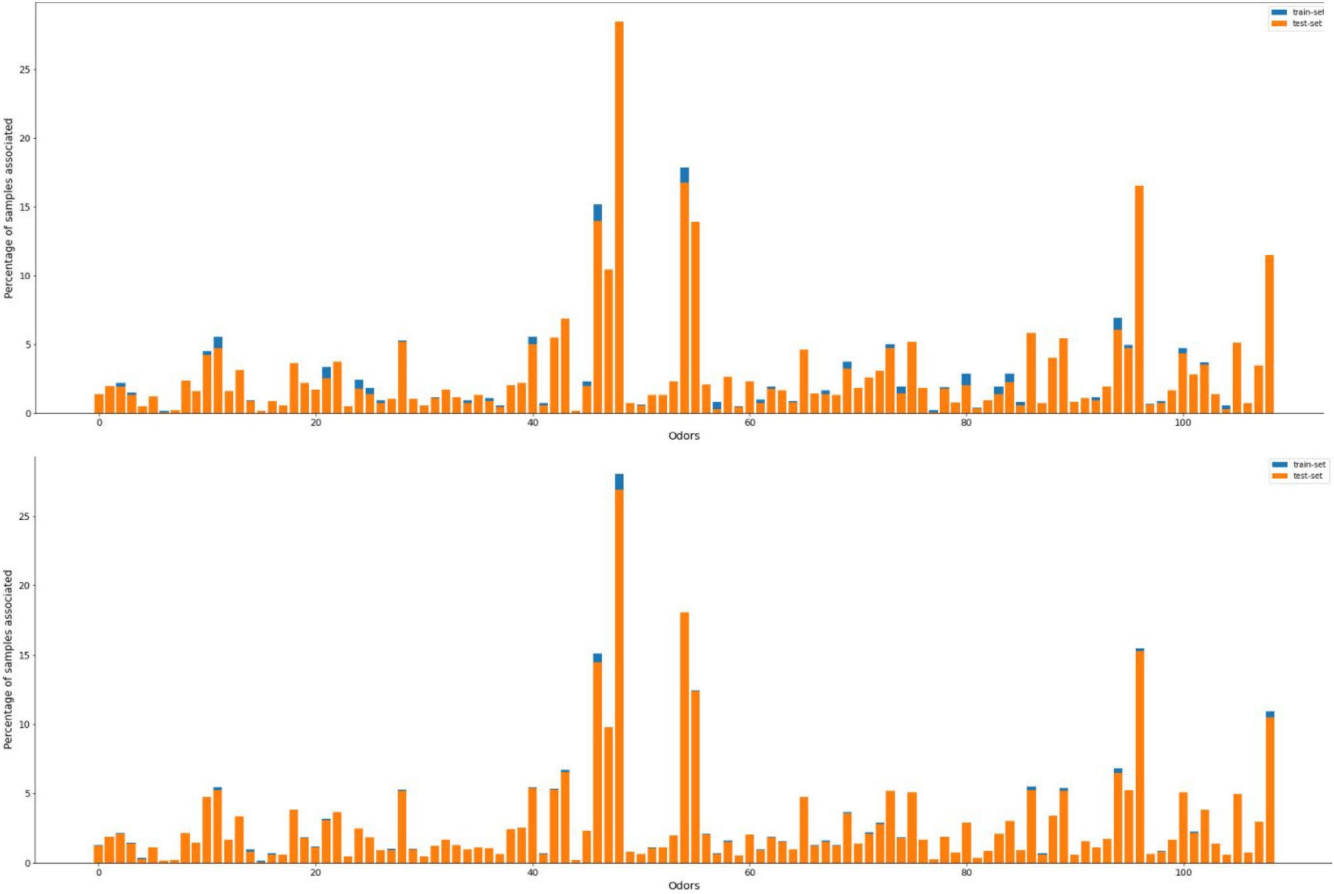
Random split versus a stratified split respectively on the data. The difference in the height of blue and orange bars represents the relative difference in the occurrence of a particular label in the test and training set. It is clearly visible that stratified sampling produces a much more representative training and test set.

To ensure that the training and test dataset have a general representation of the data, we used iterative stratified sampling instead of random sampling while splitting our dataset by importing the iterative_train_test_split class from the scikit-multilearn library as the random sampling resulted in completely omitting the fennel odor descriptor adding to the already skewed label imbalance (Fig. 6).

**Figure 7:**
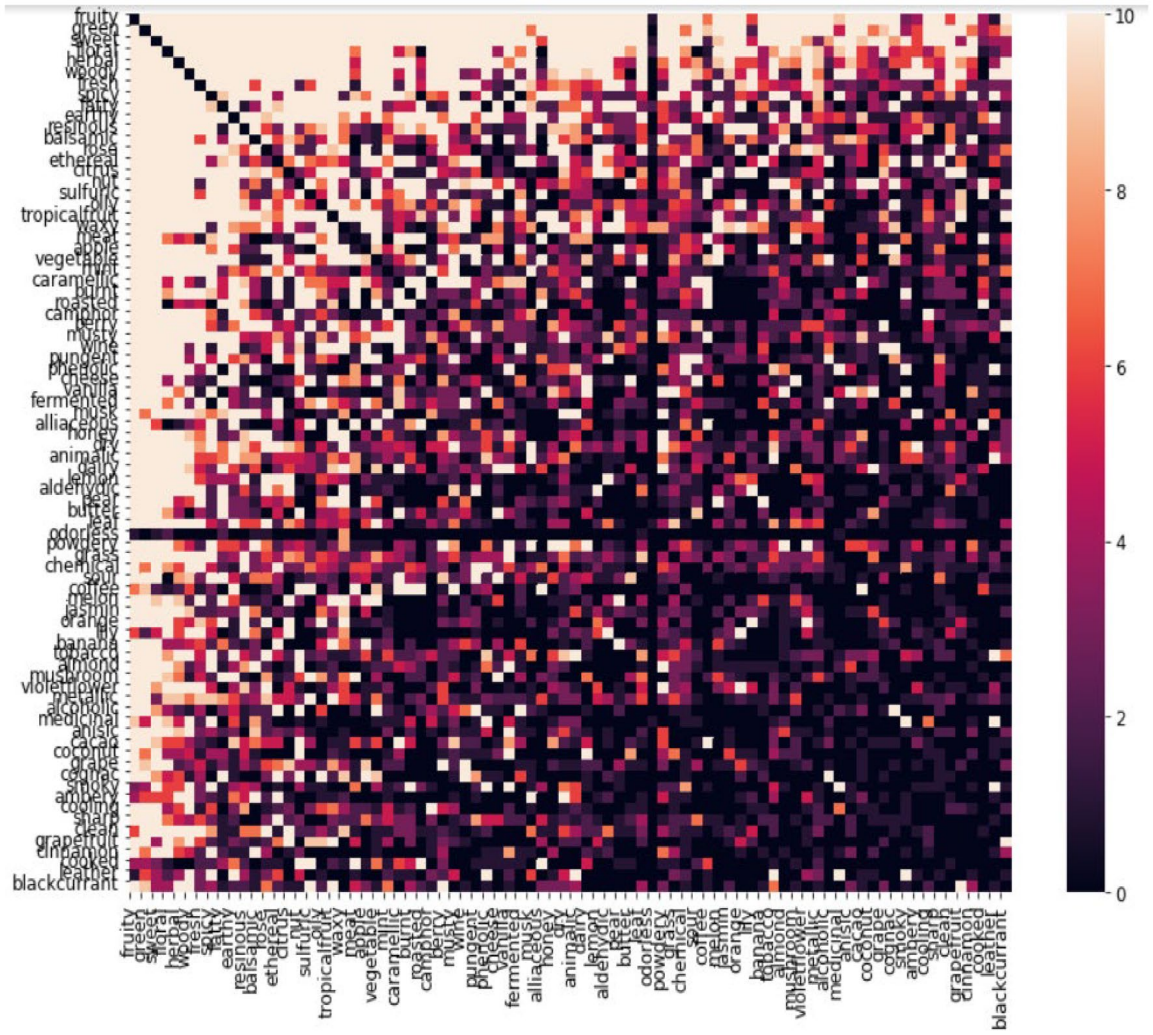
Heat map of our co-occurrence matrix truncated to top 80 odor.

The construction of the co-occurrence matrix whose heatmap is shown in Fig. 7, revealed that green and fruity labels co-occurring 518 times and sweet and fruity labels co-occurring 433 times are the top 2 frequently occurring label pairs (Table 2). That means of all the molecules having a fruity odor 25% of them also have a green odor. Conversely, 40% of all molecules having a green odor also have a fruity odor. This follows suit with the general observation with MLDs that there usually exists some label correlation.

**Table 2:** Top 20 odor associations labels. The darker the intensity of a square, the lesser the magnitude of co-occurrence count and correlation between the two labels.

Label pairs	Co-occurence count
Green-fruity	518
Fruity-sweet	433
Fruity-floral	358
Fruity-herbal	317
Sweet-floral	313
Fresh-fruity	255
Herbal-green	217
Green-flora	216
Herbal-floral	205
Green-sweet	134
Fruity-apple	174
Fruity-ethereal	174
Floral-fresh	165
Woody-floral	162
Fatty-fruity	160
Woody-fruity	159
Fresh-herbal	158
Woody-herbal	149
Herbal-sweet	141
Fruity-tropicalfruit	136

**Figure 8:**
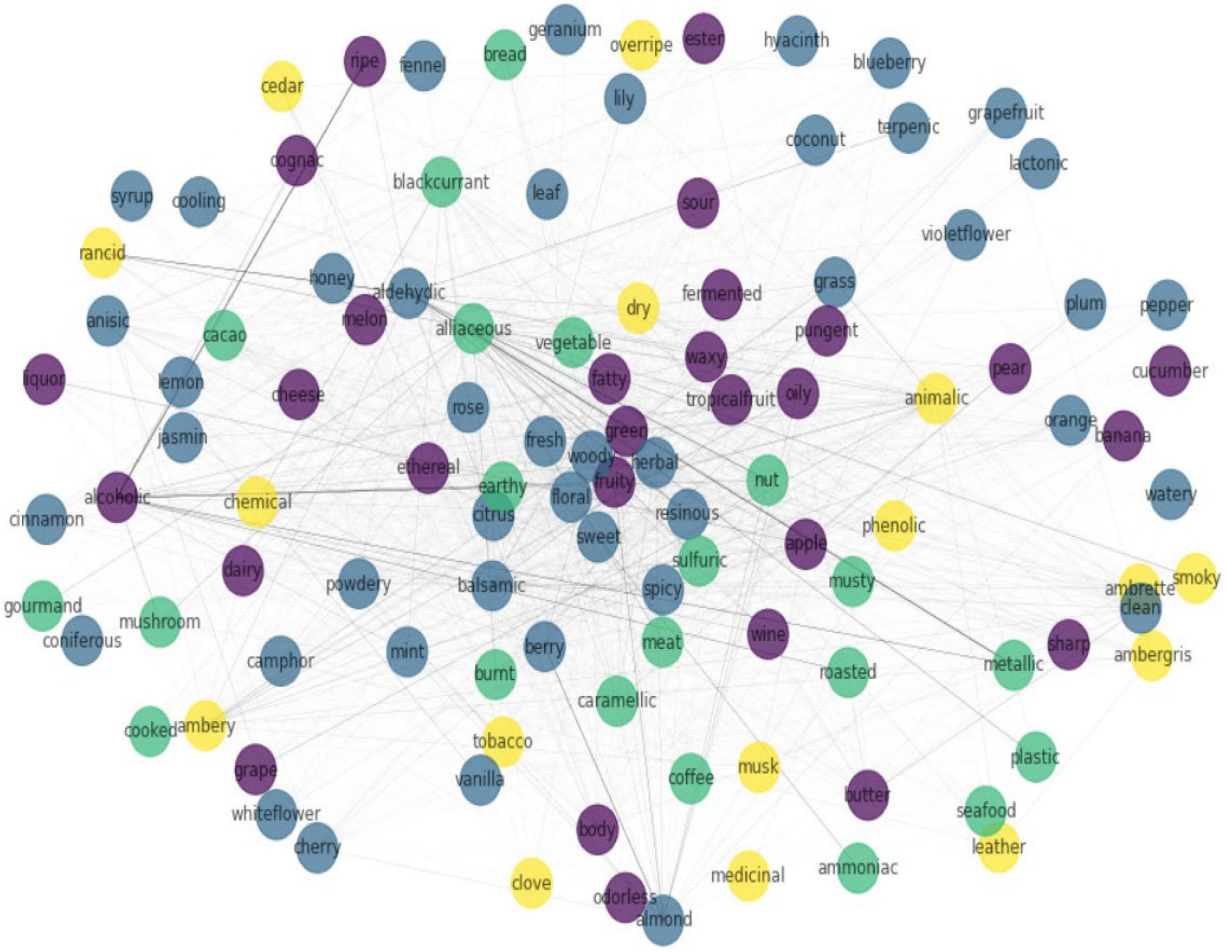
Graph constructed on the basis of Louvain method for community detection in networks. Nodes with the same colour belong to the same community.

Louvain community detection reveals the correlation between larger length label sets and it was observed that some of the groupings obtained by running the algorithm can be corroborated by common sense; for example, medicinal, phenolic, and chemical being in the same community intuitively seems right. The same is true for odor labels like fruity, apple, pear, banana, tropical fruit, melon, and grape. At the same time, some odor labels occur in communities one would not expect them to, for example, food with plastic, honey with lemon, etc. The network graph confirms that there exists some global correlation between labels. As seen in Fig. 8 the algorithm uncovered 4 clusters among a total of 109 odor labels. This approach was based on a measure called modularity, which tries to maximize the difference between the actual number of edges in a community and the expected number of edges in the community.

The cardinality of our dataset (mean number of labels per sample) is three, suggesting the multilabel-ness of our data is typical (most MLDs are in the {1, 5} interval) and the label density (cardinality divided by the number of labels) is 0.02846 (most MLDs have density values below 0.1). This value is useful to know how sparse is the labels sets in the MLD. Higher density values denote label sets with more active labels than the lower ones.

**Figure 9:**
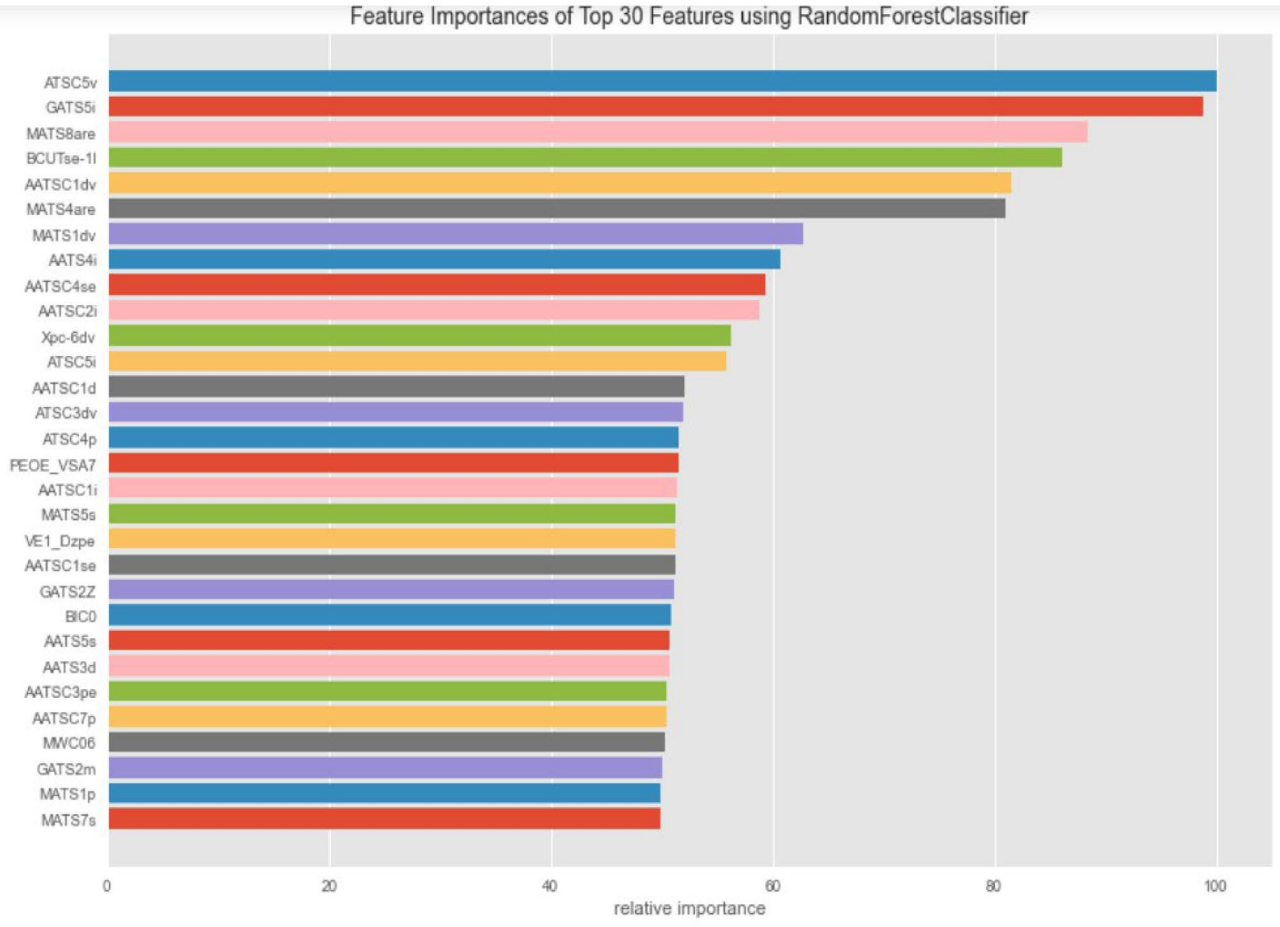
Feature importance based on closeness to the root node of decision trees in the forest computed on mordred featurization.

Figure 9 shows the feature importance which was generated with random forests to get an idea of the features which contribute most significantly to the odor prediction task. Centered moreau-broto autocorrelation of lag 5 weighted by van der Waals volume (ATSC5v) and Geary coefficient of lag 5 weighted by ionization potential (GATS5i) are the top two Mordred features of importance, and further study into these might provide us with some insight into the kind of structural fragments or their relative spatial positions that result in imparting a particular odor to molecules.

Both of these two descriptors are spatial autocorrelation descriptors which in general explain how the considered property is distributed along the topological structure of the molecule. Representing the molecule as a graph with atoms at the vertices and bonds as the edges, both the auto descriptors consider a certain molecular property (e.g. atomic masses, atomic van der Waals volumes, atomic Sanderson electronegativities, atomic polarizabilities, etc.) distribution between pair wise atoms in the molecule at a certain topological distance (smallest number of interconnecting bonds between the two atoms).

Our model performance evaluated on micro averaged scores for each featurization revealed that the binary relevance model trained on Daylight fingeprints yielded the best F1_score. It is also worth mentioning that Binary relevance produced somewhat comparable F1_scores for mordred and Morgan featurizations with classifier chains and superior F1_scores with random forest models.

It is worthwhile noting that although there is a label correlation between our odor labels, we got better model performance from binary relevance which discards these correlations as opposed to classifier chains which take them into account, as evident from Table 3. Fivefold cross validation was carried out on the training set in order to further authenticate the scores shown in Table 4. One possible explanation for this contradiction might lie in the ordering of target labels which we have taken to be random. Because the models in each chain are arranged randomly there is significant variation in performance among the chains. Presumably, there is an optimal ordering of the classes in a chain that will yield the best performance. However, we do not know that ordering a priori.

**Table 3:** Showing micro averaged precision, recall, and F1 test scores for each featurization and model approach.

	Random forest			Binary relevance			Classifier chains		
	F1 score	Precision	Recall	F1 score	Precision	Recall	F1 score	Precision	Recall
Mordred features	0.2944	0.3973	0.2338	0.3454	0.3938	0.3075	0.3246	0.4087	0.2692
Morgen fingerprint	0.2874	0.3850	0.2292	0.3351	0.3778	0.3011	0.3191	0.3963	0.2671
Daylight fingerprint	0.3221	0.3757	0.2819	0.3523	0.3563	0.3483	0.3287	0.3745	0.2930

**Table 4:** Showing micro averaged precision, recall, and F1cross-validation scores for each featurization and model approach.

	Random forest			Binary relevance			Classifier chains		
	F1 score	Precision	Recall	F1 score	Precision	Recall	F1 score	Precision	Recall
Mordred features	0.2576	0.3796	0.1950	0.3151	0.3865	0.2661	0.3028	0.4042	0.2422
Morgen fingerprint	0.2604	0.3739	0.1998	0.3044	0.3651	0.2610	0.2952	0.3889	0.2379
Daylight fingerprint	0.3027	0.3671	0.2576	0.3319	0.3520	0.3141	0.3191	0.3795	0.2753

We also computed label-wise scores (Table 5) and observed that the top 15 F1_scores generally belonged to labels that had a higher percentage of associated samples in our dataset (mean percentage of samples associated with top 15 labels = 3.553), although there were exceptions to it like fennel (0.122 percentage) and ammoniac (0.176 percentage).

**Table 5:** Bottom 15 (from ‘clean' to ‘syrup') and top 15 (from ‘ammoniac' to ‘balsamic') label-wise F1_scores along with their precision and recall scores. Percentage of samples for labels was calculated on the entire dataset and not only the test set.

	Precision_score	Recall_score	F1_score	Percentage_of_samples
Clean	0.043478	0.066667	0.052632	1.017087
Sharp	0.076.923	0.041667	0.054054	1.057771
Chemical	0.080000	0.071429	0.075472	1.695145
Rancid	0.111111	0.071429	0.086957	0.745864
Liquor	0.125000	0.066667	0.086957	0.840792
Whiteflower	0.090909	0.090909	0.090909	0.610252
Pungent	0.114286	0.086957	0.098765	2.739354
Ripe	0.125000	0.111111	0.117647	0.650936
Metallic	0.120000	0.157895	0.136364	1.288310
Dry	0.185185	0.128205	0.151515	2.061296
Lactonic	0.111111	0.250000	0.153846	0.488202
Cedar	0.200000	0.142857	0.166667	0.474641
Plum	0.181818	0.153846	0.166667	0.772986
Ambergris	0.142857	0.250000	0.181818	0.325468
Syrup	0.200000	0.200000	0.200000	0.837375
Ammoniac	0.666667	0.666667	0.666667	0.176295
Mint	0.500000	0.629630	0.557377	3.647952
Fruity	0.552163	0.529268	0.540473	27.800380
Honey	0.531250	0.548387	0.539683	2.074858
Mushroom	0.424242	0.666667	0.515513	1.383238
Fennel	1.000000	0.333333	0.500000	0.122050
Anisic	0.555556	0.454545	0.500000	1.206943
Alcoholic	0.476190	0.528318	0.500000	1.288310
Lily	0.500000	0.478261	0.488889	1.545972
Grapefruit	0.583333	0.411765	0.482759	0.976404
Jasmin	0.458333	0.478261	0.468085	1.586658
Musk	0.404762	0.531250	0.459459	2.183347
Vanilla	0.466667	0.424242	0.444444	2.224030
Coffee	0.500000	0.400000	0.444444	1.640900
Balsamic	0.465753	0.425000	0.444444	5.438025

In contrast, the bottom 15 F1_scores generally belonged to labels that had a lower percentage of associated samples (mean percentage of samples associated with bottom 15 labels = 1.027).From this, it is inferred that our model performs better on frequent labels than infrequent labels.

## Conclusion

We assembled a novel and large dataset of expertly labelled odorants and applied multi-label classification techniques to predict the relationship between a molecule’s structure and its smell. We achieved close to state-of-the-art results obtained using GNN’s y^4^ on this QSOR task, employing multi-label classification techniques, and further demonstrated the label correlations that occur in our label space. Finally, we evaluated labels for which our best performing model is a weak learner and others for which it performs well.

## Electronic supplementary material

Below is the link to the electronic supplementary material.

Supplementary Information 1.

Supplementary Information 2.

## Data Availability

All data generated or analysed during this study are included in this published article and its supplementary information files.
