# Supplementary material for: Predicting odor from molecular structure: a multi-label classification approach
Source: Sci Rep. 2022 Aug 16;12:13863. doi: 10.1038/s41598-022-18086-y (PMC9381526; doi:10.1038/s41598-022-18086-y)
Supplement: Supplementary file 2 — Supplementary Information 2. [file 41598_2022_18086_MOESM2_ESM.docx]

**A Review of Machine Learning Approaches to Predicting**

**Molecular Odor in the Context of Multi-Label Classification**

Kushagra Saini^1^, V. Ramanathan^2*^

1 Department of Chemical Engineering, Indian Institute of Technology (Banaras Hindu University) Varanasi – 221005 (UP), India.

2 Department of Chemistry, Indian Institute of Technology (Banaras Hindu University) Varanasi – 221005 (UP), India.

*Author to whom correspondence be addressed at vraman.chy@iitbhu.ac.in

**SUPPORTING INFORMATION**

Mordred featurization produced a total of 1521 features, out of which 911 features had at least one missing value. We dropped feature columns with more than 40 % missing values, which reduced the number of features to 1436. The remaining missing values were imputed using the mean strategy by a KNN imputer.

Reducing the number of Dimensions was attempted using PCA, but even while retaining 99.99 % variance, the model's f1_score dropped by up to 0.4. This is a drastic drop given the model performance for the task was very low to begin with, so we opted not to transform our data using PCA.

Expanding the dataset was attempted using APIs of online chemical databases like PubChem, ChemSpider etc. But due to very poor quality of odor vocabulary and the rarity of odor description being available for molecules in the database, we stuck with our expertly labeled odor dataset.

Fingerprints were generated using the openly licensed RDkit tool, which is popularly used in cheminformatics. It was also used to visualize our Smile strings.

Iterative stratified splitting was done using the scikit-multilearn library, and further, a custom iterative stratified splitter was passed during cross-validation.

Networkx and scikit-multilearn libraries were used to visualize our graph communities during community detection.


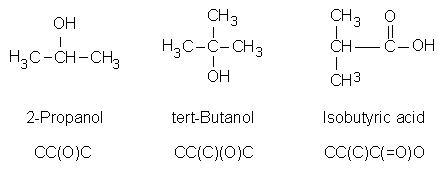

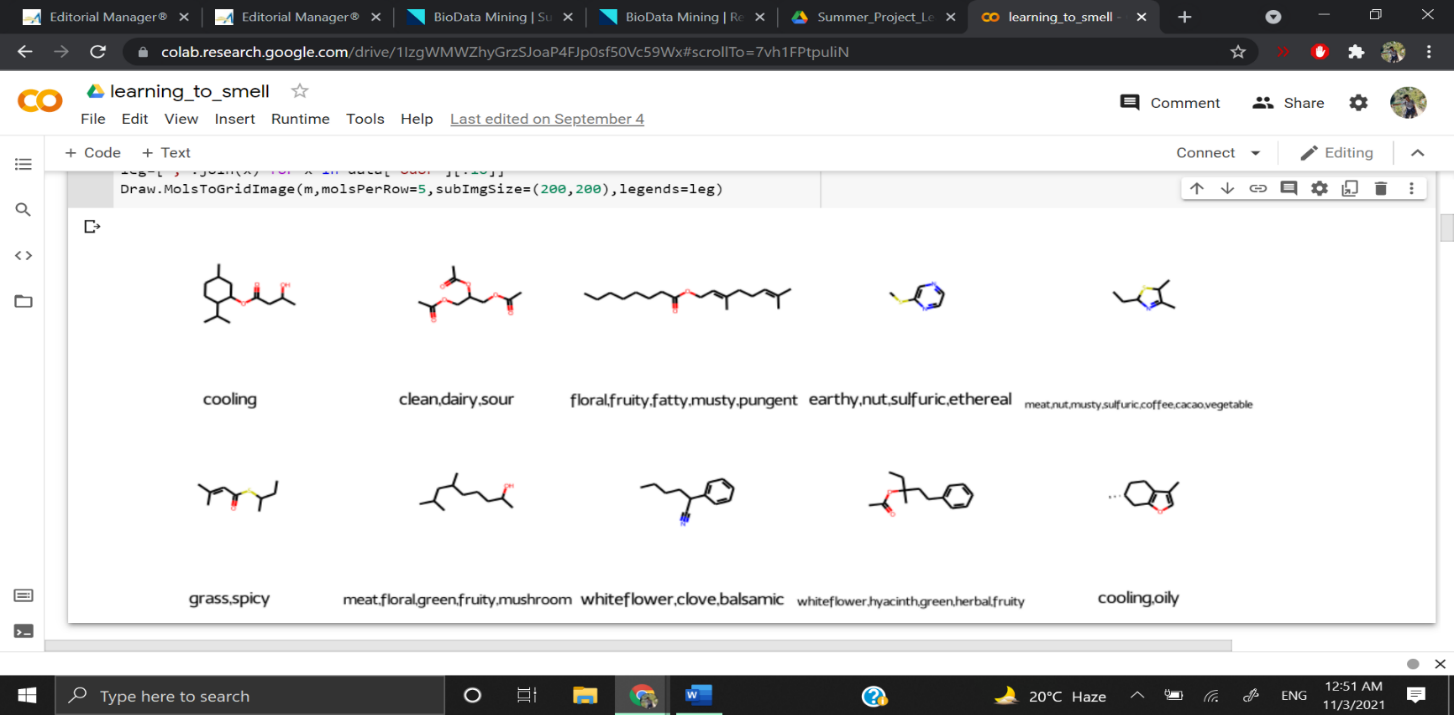


**Fig. S1**  Smile notation for branched chains with their structures **Fig. S2** Molecules generated and visualized from

smile strings in the dataset

SMILE (simplified molecular-input line-entry system) format, which is a specification in the form of a line notation for describing the structure of chemical species using short ASCII strings. This encoding into a string of characters is done to make the data machine-readable. A unique molecular structure can be reproduced from each smile string.

For ease of data visualization and exploration, we chose only to retain Firmenich’s vocabulary, thus dropping labels from the PMP database which were non-intersecting.

Mordred Featurization: - A molecular descriptor is defined as the final result of a logical and mathematical procedure, which transforms chemical information encoded within a symbolic representation of a molecule into a useful number or the result of some standardized experiment. Various molecular-descriptor-calculation software programs have been developed- Mordred, Dragon, and ChemoPy, to name a few.. It computes thousands of handcrafted features like number of carbon atoms, number of hydrogen atoms, ring count, etc, all of which transcribe the information about our molecule.

Fingerprinting: - The molecular fingerprint is just another way of numerically representing a molecule. The bit-like patterns generated by the fingerprint indicate the absence or presence of certain substructures/fragments within a molecule. A molecular fingerprint characterizes the pattern, but the meaning of any particular bit is not well defined.

Molecular fingerprints represent a set of features derived from the structure of a molecule. The particular features calculated from the structure can be quite arbitrary and depend on the topology of the chemical graph or even a 3D conformation. Different fingerprint schemes emphasize different molecular attributes according to the design philosophy of the fingerprint system. The fundamental idea is to encapsulate certain properties directly or indirectly in the fingerprint and then use the fingerprint as a surrogate for the chemical structure.

The fingerprinting algorithm examines the molecule and generates fragments iteratively. Each fragment serves as a seed to a pseudo-random number generator (it is "hashed"), the output of which is a set of bits (0’s and 1’s, typically 4 or 5 bits per fragment); the set of bits thus produced is added (with a logical OR) to the fingerprint.

Types of fingerprints are differentiated by the manner of fragment generation: -

a) Path-Based Fingerprinting(daylight)- Fragments of the molecule are generated by following a path (usually linear) up to a certain number of bonds within the molecule.

b) Morgan Fingerprinting(circular) - Instead of linearly searching along each bond to generate fragments, morgan fingerprints generate fragments radially while varying the radius in each iteration.


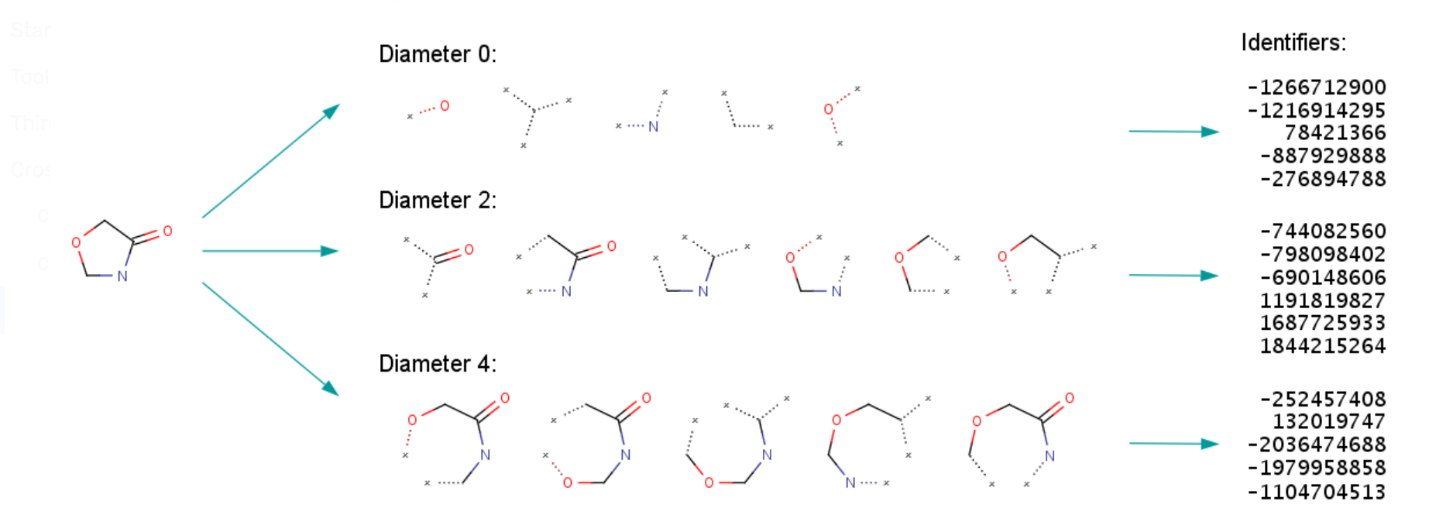


**Fig. S3**  Morgan fingerprinting scheme in action with a varying radius of 0,1,2. All configurations of the 0^th^ radius are fed to a pseudo number generator which outputs the block of identifiers. These are further hashed to give a bit string. This is done for every radius, and the output is concatenated to the fingerprint

Multi-Label classification has proven to be very useful in fields like text categorization, Analysing protein properties and gene expression, and labeling of multimedia resources. The main difference between traditional and multi-label classification is in the output expected from trained models. Where a traditional classifier will return only one value, a multi-label one has to produce a vector of output values.

Problem transformation-based multi-label classification algorithms aim to convert the original dataset into one or more simpler datasets that can be delivered to traditional classification algorithms, while algorithmic adaptation techniques rely on modifying existing classification algorithms. The changes that must be introduced in the algorithms can be quite simple or really difficult, depending on the nature of the original method and also our data.

With the IRLbl metric, it is possible to know the imbalance level of one specific label. This is computed as the proportion between the number of appearances of the most common label and the considered label. Usually, a global assessment of the imbalance in the MLD is desired. This metric, named MeanIR, is calculated by averaging the IRLbl of all labels

**List of abbreviations-**

1)QSOR- Quantitative Structure-Odor Relationship.

2)QSAR- Quantitative Structure-Activity Relationships

3)MLD’s- Multi-Label Datasets

4) SMILE - simplified molecular-input line-entry system

**Data and Software Availability**

Data access: -

This publication is supported by multiple datasets, means of access to which are mentioned below.

PMP LEFFINGWELL DATASET: -

The molecular odor data can be found in “Zenodo” repository with the identifier 10.5281/zenodo.4085098 and are available from the corresponding author upon reasonable request. [<https://zenodo.org/record/4085098>]

FIRMENICH DATASET: -

The dataset is available from the resources tab of “Learning to Smell challenge” hosted on AIcrowd platform(<https://www.aicrowd.com/challenges/learning-to-smell/dataset_files>), and is owned by Firmenich (https://www.firmenich.com/) but restrictions apply to the use of these data under license from Firmenich.

INTEGRATED DATASET: -

Formed after combining the above two datasets and applying the pre-processing steps mentioned in the paper- on which the findings of this publication rest- is provided as supplementary material accompanying this paper.

Software access: -

Mordred molecular descriptor calculator was used for generating handcrafted Mordred features for our molecules( <https://pypi.org/project/mordred/> )[version 1.2.0]

Morgan and path-based fingerprints were generated using Rdkit Toolkit ([https://pypi.org/project/rdkit-pypi/](https://pypi.org/project/rdkit-pypi/%20) ) [version 2021.09.4] and this was also used for visualizing smile strings.

Fuzzywuzzy package[0.18.0] (<https://pypi.org/project/fuzzywuzzy/>) was used to find similar odor labels from two separate odor datasets so as to establish a consistent odor vocabulary

All machine learning models used during the course of this project have been imported from the scikit-learn open-source library(<https://github.com/scikit-learn/scikit-learn>)

Iterative-stratification package was used for stratified splitting into training and test set(<https://pypi.org/project/iterative-stratification/>) [version 0.1.7]
